# More than a drop of blood: the paradigm shift of a large-volume capillary sample for point-of-care clinical diagnostics

**DOI:** 10.1128/jcm.00895-25

**Published:** 2026-03-30

**Authors:** Gregory L. Damhorst, Anuradha Rao, Nira R. Pollock

**Affiliations:** 1 Division of Infectious Diseases, Emory University School of Medicine 12239, Atlanta, Georgia, USA; 2 Department of Pediatrics, Emory University School of Medicine 12239, Atlanta, Georgia, USA; 3 Department of Laboratory Medicine, Boston Children's Hospital 1862, Boston, Massachusetts, USA

**Keywords:** capillary, lancet, fingerstick, blood collection, point-of-care, diagnostics

## Abstract

The validation of a large-volume fingerstick sample for the newly Food and Drug Administration-authorized Xpert HCV test has created an important precedent for the use of a large-volume capillary blood sample for point-of-care (POC) testing. This advance opens the door to the development and deployment of a wide range of POC tests utilizing large-volume capillary samples, including tests that require larger sample volumes for detection of low-concentration analytes. Multiple tools for production and collection of large-volume capillary samples now exist, including many tools designed for yielding blood from capillaries in the upper arm. Most of these tools yield blood that demonstrates excellent concordance with venous blood on testing across a range of analytes, though analytes like potassium and platelets remain challenging for the arm-based tools, and data for molecular analytes are limited overall. Tools capable of separating liquid plasma from large-volume capillary whole blood samples for POC testing remain an elusive but critical goal. Understanding the regulatory expectations for pairing large-volume sample production tools and collection containers with POC testing devices will facilitate both uptake of large-volume collection strategies and POC blood test development, particularly because allowing multiple POC tests to be performed from one large-volume capillary sample would substantially improve the patient experience. Large-volume capillary blood collection is a new frontier key to the future of POC diagnostics.

## INTRODUCTION

The recent granting of Food and Drug Administration (FDA) marketing authorization to the first point-of-care (POC) nucleic acid amplification test for the detection of hepatitis C virus (HCV) RNA in blood (1) represents an exciting paradigm shift for diagnosis of HCV infection. The newly authorized Xpert HCV test and GeneXpert Xpress System (Cepheid, Sunnyvale, CA) utilizes 100 µL of fingerstick (FS) blood and provides a result in less than 1 hour. This novel test, enabling diagnosis and prescription of treatment for HCV infection at a single visit, marks a major step towards the goal of HCV elimination (2). The Xpert HCV clinical trial, data from which were recently summarized by Havens (3), comprehensively evaluated the clinical performance of this test. Nested within this new approach to HCV diagnosis is an additional paradigm shift that deserves its own mention: the use of a large-volume capillary blood sample (defined here as ≥250 μL) for testing performed at the POC.

## TOOLS AVAILABLE OR IN DEVELOPMENT FOR LARGE-VOLUME CAPILLARY BLOOD PRODUCTION AND COLLECTION

### The important precedent set by Xpert HCV: large-volume fingerstick blood collection for POC testing

Previously, the volume of FS blood used for FDA-cleared POC tests has typically ranged from 1 to 2 µL (e.g., glucometers used in hospital settings [4]; some hemoglobin (HgB) measurements [5]) to 50 µL (e.g., HIV antigen/antibody testing [6]). The CE-marked Xpert test (Xpert HCV VL Fingerstick) (7), which preceded the U.S. Xpert HCV test development process, uses a 100 µL EDTA whole blood FS sample collected using a specific capillary tube with a built-in plunger (Minivette POCT; Sarstedt, Newton, NC [8]). While this tool is cleared for FS blood collection in the U.S.A. with volumes ranging from 10 to 200 µL and a 20 µL version is used in at least one POC test with Emergency Use Authorization (LumiraDx SARS-CoV-2 Ab Test; Roche, Indianapolis, IN) (9), studies had observed that Xpert HCV VL testing using the 100 µL pipette generated frequent invalid results related to insufficient blood volume (10, 11), suggesting that an alternative, larger-volume approach to FS collection could have value. The Xpert HCV test with FDA marketing authorization utilizes a K2EDTA microtainer (BD 365974) with a 250–500 µL volume range (once collected into this microtainer, 100 µL of FS blood is transferred into the Xpert cartridge using a transfer pipette supplied with the Xpert cartridge). While the EDTA microtainer is FDA-cleared for collection of capillary blood for hematologic testing (12), it had not previously been used for molecular testing nor for testing performed at the POC.

The Xpert HCV Instructions for Use [IFU (13)] indicate that reliable collection of 250–500 µL blood into the microtainer requires not only a high-flow lancet (2 mm minimum depth) but also some initial operator guidance. The Xpert HCV test uniquely provides FS sample collection instructions through a video made available via a QR code embedded within the IFU (13) and Quick Reference Instructions, which must be viewed prior to the first collection attempt. These instructions include details key to volume yield, such as warming the patients’ hands, having the patient exercise their hand, applying firm pressure when depressing the lancet, and gentle fingertip massage (without “milking,” as this is known to cause hemolysis). Additional instructions educate the user to tap the tube against a surface to avoid coagulation and to collect the proper volume. During the trial, it was observed that with this guidance, blood collection by this method was ultimately reliable (though as shown in the video described above, it takes a few minutes to collect at least 250 µL) (3). Notably, the study population included people who experience homelessness and people who inject drugs, generating specific blood collection challenges (collection of FS samples in the context of calloused fingertips and venipuncture samples in the context of poor venous access). The study showed that frequencies of unsuccessful collection for both FS and venous sample types were similar, supporting the high-volume FS approach for the HCV POC test. While these data are encouraging, real-world sample collection data outside of the clinical study setting are yet to be gathered.

Notably, BD has newly developed the BD MiniDraw Capillary Blood Collection System (Becton, Dickinson and Company, Franklin Lakes, NJ) (14) for use with the BD Microtainer that facilitates consistent gentle squeezing of the patient’s fingertip to release blood and collect it in an attached microtainer. This system has FDA 510(k) clearance for selected chemistry analytes (15) and HgB and hematocrit (HCT) when analyzed on Sysmex XN-Series systems (Sysmex, Kobe, Japan) (16), but not yet for molecular testing, and is discussed in further detail below. While this tool may increase the consistency of large-volume FS, it adds expense. Currently, there are no other FDA-cleared large-volume production/collection devices utilizing FS besides the BD MiniDraw System.

### Arm-based devices for production of large-volume capillary blood samples

Over the past few years, multiple novel devices have been developed that can facilitate production of large volumes of capillary whole blood from the upper arm, offering the promise of simpler and less painful blood production compared to traditional fingerstick. Table 1 summarizes large-volume production devices that have been cleared by the FDA. The arm-based devices use one or more short lancets or needle blades to puncture the skin, may or may not use vacuum/suction function, and deliver capillary blood into a variety of tubes that can subsequently be removed and capped (17, 18). Other similar devices in development but not yet cleared by the FDA (18, 19) have not been included in Table 1.

**TABLE 1:** Summary of FDA-cleared tools for large-volume (≥250 µL) capillary blood production or collection^*a*^^,^^*b*^^,^^*c*^

Device name	Ref	Type of use	Predicate	Site	Sampling mechanism	Intended use
Tasso+	(20)	Prescription	Tianjin Huahong Technology Safety Lancet	Upper arm	Single lancet	Obtaining microliter capillary whole blood samples
TAP Lancet	(21)	Prescription	Tasso+	Upper arm	1 mm lancet and vacuum	Producing microliter capillary whole blood samples
RedDrop ONE (One)	(22)	Prescription	TAP Lancet	Upper arm	2 mm lancet array	Producing microliter capillary whole blood samples
LetsGetChecked Impress	(23)	Prescription	TAP Lancet	Upper arm	Dual lancets and vacuum	Producing microliter capillary whole blood samples
NanoDrop Lancet	(24)	Over the counter	Suzhou Zhenwu Medical Co., Ltd., Safety Lancet	Upper arm	Dual needle blades and vacuum	Obtaining a capillary blood sample
BD MiniDraw Capillary Blood Collection System with BD MiniDraw SST Capillary Blood Collection Tube	(15)	Prescription	BD Microtainers	Finger	BD Microtainer Contact-Activated Lancet (K223243)	Sample collection used in the measurement of ALKP, ALT, Na, Cl, ALB, BUN, Ca, CREAT, TBIL, TP, HDL, LDL, CHOL, and TRIG from individuals aged 18 years and older
BD MiniDraw Capillary Blood Collection System with BD MiniDraw H&H Capillary Blood Collection Tube	(16)	Prescription	BD Microtainers	Finger	BD Microtainer Contact-Activated Lancet (K223243)	Sample collection used in the measurement of HgB and HCT from individuals aged 18 years and older when analyzed on Sysmex XN-Series systems

^*a*^ Other than the BD MiniDraw System, these devices are not cleared for sample collection nor to facilitate the analysis of specific analytes, nor is patient age specified.

^*b*^ In contrast, the BD MiniDraw System is cleared for the analytes listed and for use in adults (≥18). The NanoDrop Lancet is unique in having been designated for over-the-counter use.

^*c*^ ALB, albumin; ALKP, alkaline phosphatase; ALT, alanine aminotransferase; BUN, blood urea nitrogen; Ca, calcium; CHOL, total cholesterol; Cl, chloride; CREAT, creatinine; HCT, hematocrit; HDL, high-density lipoprotein; HgB, hemoglobin; LDL, low-density lipoprotein; Na, sodium; TBIL, total bilirubin; TP, total protein; TRIG, triglyceride.

Regulatory approval summaries available on the FDA website highlight the distinction between blood “production” and “collection” for these devices. Only the BD MiniDraw fingerstick systems present data in regulatory documents to support equivalency of analyte measurements compared to a venous sample (15, 16). In contrast, the arm-based devices Tasso+ (Tasso, Inc., Seattle, WA), TAP Lancet (YourBio Health, Medford, MA), RedDrop ONE (RedDrop Dx, Fort Collins, CO), LetsGetChecked Impress (LetsGetChecked, Atlanta, GA), and NanoDrop Lancet (Drawbridge Health, Inc., New York, NY) claim an indication for “producing” or “obtaining” microliter capillary whole blood samples and further specify that they do not “collect or transport” such samples (20–24). Accordingly, the predicate devices employed in filing for 510(k) clearance of these arm-based devices are lancet devices, while the BD MiniDraw System (which requires a specific fingerstick lancet) uses BD Microtainers as predicate. Kits including both the Tasso+ and a BD Microtainer tube are sold for Research Use Only (25), consistent with the observation that the combination has not yet been cleared by the FDA for production and collection. This distinction highlights the regulatory processes still needed to ultimately deploy these newer blood production and collection devices in clinical applications, as discussed further below.

Although the scope of cleared use remains limited, less painful and more user-friendly approaches to producing capillary blood open the door to broader use of these devices for blood analysis at the POC or even at home. Notably, one recently cleared device, the NanoDrop Lancet (Drawbridge Health, Inc.), has over-the-counter designation to obtain a capillary blood sample (24), while the others in Table 1 remain prescription use only.

### Large-volume capillary sample quality

Though FDA clearance of the arm-based capillary blood production devices does not encompass blood collection for the analysis of specific parameters, multiple studies have examined the quality of capillary blood samples obtained using these devices, and by extension their potential clinical utility. The largest quantity of published data is for the Tasso+ device. Few studies other than the Xpert HCV study (3) have examined measurements of viral analytes in large-volume capillary blood, one exception being a robust study of CMV DNA in blood yielded with the Tasso+ (26). In this study, solid organ transplant recipients with CMV DNAemia were recruited to either self-collect or have a research coordinator collect capillary blood using the Tasso+ for CMV DNA monitoring (26). The arm was warmed with a thermal pad and the device was placed for 10 minutes or until 500 µL had been collected. Whole blood was centrifuged and plasma removed and frozen until batched analysis (samples were thawed and diluted with PBS to a final volume of 700 uL for testing) on the Abbott M2000 extraction and qPCR platforms. The study was unable to collect capillary blood due to device failures in 2 of 49 collection attempts from 30 participants. The study yielded 91% (40 of 44 sample pairs) categorical agreement between capillary and venous blood. Among discordant specimens, CMV DNA was not detected in four capillary samples, one of which had a paired venous sample below the limit of quantification but not below the limit of detection, and three where the venous viral load was above the limit of quantification (viral loads for these are presented as median [IQR] 116 (98–151) IU/mL). Linear regression analysis of samples that produced quantifiable results for both capillary and venous samples showed an *R*^2^ of 0.991 and a concordance correlation coefficient of 0.988. Tasso+ was also studied in the production of blood samples for HIV and syphilis testing (27).

The Tasso-SST, a Tasso device that appears to be similar to the FDA-cleared Tasso+ but has been described as having a different lancet mechanism (28), has also been evaluated in a small number of studies. One study examined self-collected capillary blood produced with the Tasso-SST device compared to venous blood in a group of patients on HIV pre-exposure prophylaxis (PrEP), suspected to have a syphilis infection, or living with HIV (29). The study reported that 5 of 21 (24%) first collection attempts resulted in insufficient sample for testing, but 100% of 27 participants who made a second attempt were able to produce enough specimen for at least one assay (HIV Ag/Ab EIA or RPR assay). One hundred percent concordance between capillary and venous blood samples was observed (7 positive and 29 negative HIV Ag/Ab EIAs). The only capillary-venous discordance among 31 participants for whom an RPR assay was performed (20 non-reactive and 11 reactive venous samples) was concluded to be a false-positive venous RPR result. Another study utilized the Tasso-SST in combination with SARS-CoV-2 serological testing (30). It is not immediately clear whether data gathered with the Tasso-SST will be identical to that gathered with the Tasso+ devices, but one study used both and observed more bleeding with the Tasso+ device, consistent with a difference in lancing mechanism (28).

In performing an analysis of measurements made on the Roche Cobas chemistry analyzer, Wickremsinhe et al. (31) examined both centrifuged Tasso+-produced specimens shipped at ambient temperatures and whole blood specimens shipped refrigerated, noting that 95% and 89% of the specimens, respectively, met the quality threshold for hemolysis to allow analysis, but capillary-venous concordance of potassium measurements was poor (31). Poor concordance with venous potassium measurements was also observed in a study of the Tasso+, RedDrop, and TAP Micro devices (32). In contrast, a study done around the same time using the Tasso+ device concluded that there was good correlation of capillary blood potassium measurements made with a novel device with venous blood potassium measurements made with a reference method (iStat POC) (33). Given the differences in study design, it is difficult to draw a conclusion about the reliability of potassium measurements in blood produced with a Tasso+ device. Evidence of poor concordance between Tasso+-produced and venipuncture specimens for platelet measurements has also been described (34). Tasso+ has also been studied for high-throughput proteomics (35) and antimicrobial therapeutic drug monitoring (36). Host RNA has been examined in some feasibility studies with the Tasso-SST device, though without direct venous comparison (37–39), but other than CMV (26), few molecular analytes have been examined.

There are relatively sparse data published on sample quality for 510(k) cleared arm-based devices other than Tasso+. TAP II was studied across 43 participants and capillary blood compared to venous blood for a complete metabolic profile and lipid profile (40). Higher levels of hemolysis were observed with TAP II-produced specimens. A negative bias in quantitative measures of creatinine was observed, and poor correlation between capillary and venous carbon dioxide, glucose, and potassium was observed (40). In another study, CBC parameters were examined using the RedDrop and Tasso+ in comparison to a venous sample for 10 participants, once again showing poor capillary-venous agreement for platelet counts with both devices (41).

As mentioned previously, the FDA clearance for the BD MiniDraw tool covers measurement of a number of specific analytes (Table 1). DiPasquale et al. (42) did a comprehensive sample equivalence study comparing results for a wide panel of chemistry and hematology analytes measured in FS blood collected with the BD MiniDraw device (into BD serum separator tube [SST] and EDTA whole blood microtainers) vs venous blood collected in standard BD vacutainers and demonstrated measurement equivalence between capillary and venous sample types across analytes, including potassium. Importantly, they demonstrated equivalence between capillary and venipuncture blood samples for platelet measurement, which has not always been straightforward for fingerstick sampling (43). Notably, they did not observe any evidence of consistent pre-analytical dilution (from mixing of interstitial fluid) of the capillary sample relative to the venous sample. The authors also hypothesize that the consistent results for blood count markers measured from the MiniDraw H&H EDTA tube may in fact be related to the large blood volume and post-collection sample mixing overcoming the drop-to-drop variation observed in collection volumes <100 µL (likely related to variable interstitial fluid contamination) (44). Collectively, these data highlight an additional advantage of large-volume capillary collection: using an aliquot of a larger-volume, well-mixed sample reduces the variability observed in measurements made from smaller drops of blood. No significant hemolysis was observed for capillary collection with the BD MiniDraw System (42), though the impact of using only trained operators for collection is unknown. Notably, despite the promising performance demonstrated in DiPasquale et al. (42), the list of FDA-cleared analytes for the BD MiniDraw System does not include platelet count, AST, or potassium, reiterating that measurements of these analytes are particularly sensitive to sample quality (43).

Arm-based devices in development do not yet appear to have solved the hemolysis challenge. For example, in an evaluation of the OnFlow device (19), 35% of samples collected with the device showed evidence of mild hemolysis, and accordingly, LDH and potassium levels each showed increased variability vs venous measurements, though the clinical implication of this variability was unclear. Recently, the RedDrop ONE, Tasso+, and TAP MicroSelect devices were compared against venous sampling and the Labcorp Comfort Draw, a silicone bulb that applies a vacuum to assist blood production from an incision made in the upper arm by a conventional lancet (32, 45). Across 41 participants, mean blood volume collection exceeded 470 µL except for the TAP device (32). Hemolysis indices (estimation of hemoglobin level in serum) observed for all blood production devices were significantly higher than those of venipuncture except for the Tasso+ device (32).

## NEW OPPORTUNITIES AFFORDED BY LARGE-VOLUME CAPILLARY BLOOD COLLECTION DEVICES

### Universal front-end sample collection

As more tools facilitating large-volume capillary blood production and collection become available and their use enters the mainstream, demonstration that the blood collected can be used for many different types of assays (hematology, chemistry, molecular, and even culture) will facilitate these tools being used in conjunction with many different POC tests. Currently, the FDA pathway for a new POC test requires that the specific blood production approach and collection container that will ultimately be used for POC testing be evaluated in the clinical trial of that POC test; accordingly, the Xpert HCV test is specifically authorized for use with a fingerstick sample produced with a traditional lancet (not the BD MiniDraw apparatus) and collected in the BD EDTA microtainer (13). Given that a large-volume capillary sample likely has sufficient volume for use in more than one POC test (below), obtaining FDA clearance for use of that large-volume sample across a broad range of analytes will directly facilitate its use with a broad range of POC assays. To maximize the benefit and cost-effectiveness of these novel large-volume production/collection tools, understanding the regulatory requirements that would broadly enable the use of blood produced and collected with these tools for POC testing will be essential, and many questions will need answers.

As noted above, other than the BD MiniDraw System, which includes a lancing device and a specific microtainer cleared for specific analytes (not including molecular testing), large-volume arm-based devices are cleared as lancing devices “intended for producing microliter capillary whole blood samples” (Table 1) and indications for use specify that the lancet “does not collect or transport such samples” (20–24). These production devices do not specify in FDA filings or product literature which blood collection container should be used and presumably will require pairing with specific microtainers for specific assays. These devices also differ in terms of their puncture and capillary blood extraction methods, which will likely impact key sample quality parameters such as hemolysis and interstitial fluid content. Consequently, the interchangeability of these lancing devices and collection containers from a regulatory perspective must be elucidated to facilitate their use in POC testing. For example, what data are needed to allow clearance of the Xpert HCV test with a capillary sample produced with an arm-based tool (as opposed to a large-volume fingerstick) and collected into the same BD EDTA microtainer? Does the answer depend at all on the specific method used by that collection tool, i.e., microneedles vs lancing, or could any arm-based device be used with the same microtainer without further validation and regulatory review? In other words, how interchangeable are the large-volume production devices from a regulatory perspective, and does this vary by analyte? Could demonstration of efficacy of a specific production device (Tasso+, TAP, RedDrop, NanoDrop, or Impress) and a specific collection tube (e.g., BD microtainer) paired with a sufficient number of analyzers be sufficient to pave the way for using the production-collection combination to produce blood for any POC device measuring the same analyte, much like the BD MiniDraw SST clearance appears to permit (15)? As another example, if a POC antigen test is cleared with a small-volume sample collected with a heparinized capillary tube, what data are needed to allow clearance of the same test with a large-volume sample collected in a heparinized microtainer (which will require use of an additional tool to transfer anticoagulated blood to the test)? The answers to these and other regulatory questions can both maximize the utility of large-volume capillary samples and expedite the development of novel POC tests designed to use them. In the meantime, broad clearance of large-volume capillary blood collection containers (like the BD microtainer) for as many types of analytes as possible (in particular, for general molecular testing) would facilitate broad use of large-volume samples for POC testing. Furthermore, additional research efforts may be able to establish reproducible compatibility of cleared capillary blood production devices with measurement of both specific analytes (e.g., potassium) and broad classes of analytes (e.g., antigens and nucleic acids for diagnosis and monitoring of infectious diseases like HIV, HCV, and HBV) for which POC testing is particularly relevant.

### Performance of multiple POC tests from one capillary sample

Currently, each POC FS test typically requires its own FS sample. As more POC tests utilizing capillary blood become available, the ability to use a large-volume sample for multiple tests (thus avoiding multiple separate fingersticks) will become even more important (46). Examples of the need for multiple POC tests run in parallel would include those being evaluated for HIV treatment (e.g., HIV test, CD4 count, liver and kidney function, and hemoglobin), suspected malaria (e.g., malaria test, G6PD test, and hemoglobin), or routine antenatal care (e.g., HIV test, syphilis test, hemoglobin, and blood typing). Notably, the practice of performing multiple POC tests at a single patient visit is common in some primary care settings in resource-limited areas (summarized in reference 46). Additionally, though high-flow lancets are available, it is challenging to collect blood from a single FS with multiple collection devices in series. The large volumes produced with the tools described here offer the potential opportunity to use one capillary sample for multiple POC tests performed in parallel, if all tests were validated for the same sample type. Importantly, use of the capillary sample for multiple tests will still require transfer of blood from the collection tube to the test with a separate tool.

### Detection of low-concentration analytes

**Fig 1:**
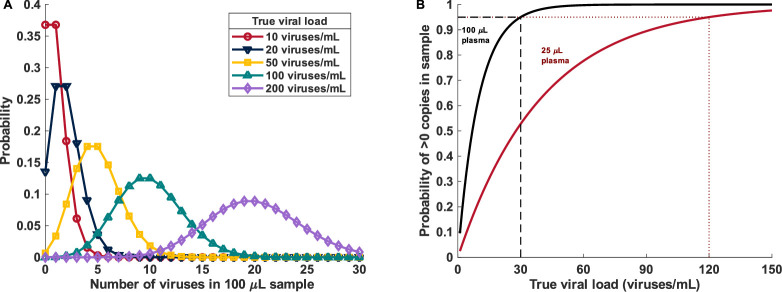
Capillary blood sampling and sensitive point-of-care molecular devices may enable increased access to blood-based assays for detection or monitoring of low-concentration analytes. (**A**) At low analyte concentrations, the probability that a discrete number of analytes (here, a hypothetical virus with one copy of nucleic acid target per virion) will be present in a small-volume sample (100 µL plasma is used here as an example) can be described by the Poisson distribution, which depends on analyte concentration (here, “true viral load”) and sample volume. Poisson statistics describe theoretical limitations of capillary sampling (in terms of analyte copies present in the sample for detection). (**B**) Probability of having one or more viruses present in a 100 or 25 µL plasma sample as a function of viral load. Annotations highlight that a true viral load of 30 viruses/mL would generate at least 1 virus in a 100 µL specimen (as might be reasonably recovered from a large-volume capillary device) 95% of the time, whereas a viral load of 120 viruses/mL would generate at least 1 virus in a 25 µL specimen (more consistent with traditional fingerstick sampling) 95% of the time. These are inherent mathematical limitations of small-volume sampling independent of the assay technology, defining minimal sample volume requirements even for highly sensitive molecular detection technologies. Importantly, approximately twice this volume of capillary whole blood would need to be collected to obtain the volumes of plasma described (e.g., ~200 µL whole blood needed to obtain 100 μL plasma).

For analytes with extremely low concentrations in blood, higher blood volumes are needed to allow analyte detection and quantification. In some cases, volumes traditionally used for fingerstick (<50 µL) may not contain enough of the analyte even for the most sensitive detection methods (e.g., nucleic acid amplification). Considering qualitative detection of a hypothetical bloodborne virus at very low concentration in blood as an example, the probability of obtaining k viruses in a discrete volume of blood is governed by the Poisson distribution (47).


$$P\left(k\right)=\;\frac{\lambda^{k}e^{-\lambda }}{k!},$$

where *λ* is determined by the true viral concentration in the sample (c) and the volume of blood sampled (V), or λ=V×c. Probabilities of a small-volume sampling containing discrete quantities of analyte are illustrated in Fig. 1A. While the Poisson law provides an understanding of how the lower limit of detection will be mathematically limited by sample volume, it also demonstrates that large-volume capillary devices enable sample volumes that should be sufficient for the detection of low concentrations (less than 100 viruses/mL) in many important POC use cases, such as HIV viral load detection and monitoring (48), while traditional fingerstick may not (Fig. 1B).

### Potential for use for home collection/testing

There is considerable interest in applying large-volume capillary blood production/collection tools to home sample collection and testing. Only the simplest and most reliable collection approaches will work in the home context, and substantial challenges remain, including reliable application of a device to one’s own arm or finger, difficulty in assessing sufficient volume yield during collection, separation of the collection tube from the device and recapping the tube, and transfer of blood out of the collection tube and onto the POC test. There has been limited experience doing this with more traditional lancet and microtainer tools. Recently, Project Home-MaDE (At-Home Microtainer and Dried Blood Spot Evaluation) aimed to determine suitability of a self-collected large-volume fingerstick using a 2 mm depth lancet and the K2EDTA BD Microtainer (365974) and compare microtainer performance to dried blood spots (49). Nearly half (27) of 57 participants did not collect sufficient volume on the first attempt, although 24 of these were able to collect sufficient specimens with a second attempt (49).

## THE NEXT FRONTIER: KEY BARRIERS TO BE CONQUERED

### Challenging analytes

As noted above, studies evaluating results for measurement of broad classes of chemistry and hematology analytes in large-volume capillary blood vs venous blood generally show promising correlation. However, there are a few outlier analytes that have consistently been particularly challenging, especially potassium and platelets (31, 34, 40, 41). Future work evaluating these large-volume capillary blood production/collection tools should include quality assurance metrics like sample rejection rates, particularly for analytes that are known to be sensitive to sample quality like platelet count, AST, and potassium (43).

### Conversion of whole blood to liquid plasma or serum for POC testing

The use of FS or arm-based tools for large-volume capillary whole blood sample collection does not currently provide a solution for POC tests using plasma or serum. For many tests, including those for analytes relevant to POC testing, plasma or serum provides a more favorable matrix for testing than whole blood (in part because hemoglobin may be inhibitory to some assays). This is particularly important for analytes that require highly sensitive/quantitative detection. In some cases, whole blood is undesirable because cellular components can confound the intended measurement. However, there are currently very few solutions for separation of capillary whole blood to generate liquid plasma or serum at POC, hindering the smooth transition of lab-based assays using plasma/serum to methodology suitable for POC use. Of the available large-volume capillary blood production/collection devices, none directly convert blood to liquid plasma (Table 1).

An obvious approach to the generation of plasma from a large-volume anticoagulated capillary sample would be to centrifuge the collection tube and remove the plasma; centrifugation would also work for the generation of serum, particularly using collection tubes with gel barriers to cleanly separate serum from clot debris. Microtainers used for collection of large-volume capillary blood samples (by FS or arm-based methods) can easily be centrifuged for generation of plasma/serum (42), but to date, there is no precedent for using a centrifuge for generation of plasma or serum at POC for waived testing (note: separation with gravity alone is time-consuming and incomplete). At the moment, centrifugation requires multiple operator steps, increasing complexity and potential risk (e.g., aerosolization, leakage, and/or exposure to infectious agents). Interestingly, work is in progress to develop a centrifuge that might be suitable for waived testing at POC. The LabCorp TrueSpin centrifuge is a class I FDA-registered, battery-operated device that can separate a microtainer sample to serum or plasma in approximately 5–10 minutes; the centrifuge does not need to be balanced by the operator and has a pre-set spin setting, facilitating use by an untrained individual (50). The centrifuge, which is not yet commercially available, has been demonstrated to produce high-quality serum from venous and capillary whole blood samples in microtainer SSTs (50); evaluation of plasma is in progress. An additional small study demonstrated that lay users could self-collect blood with a Tasso+ device, separate it to serum using the TrueSpin centrifuge, and ship it to LabCorp via overnight shipping (51), suggesting that such a workflow may also be feasible for POC waived testing. Of note, use of a centrifuge for generation of plasma for POC use would likely require new development of EDTA microtainers with gel barriers (currently, only LiHep microtainers with gel barriers exist) to facilitate easy removal of plasma.

Given the current absence of a clear pathway to use of centrifugation for waived POC testing, alternative approaches are needed for generation of plasma at POC. While it is relatively straightforward to use filtration membranes to deliver small volumes of plasma directly to integrated tests on paper (52), it is a different challenge to generate liquid plasma from capillary blood in order to provide a generic “universal front-end” solution for POC tests utilizing plasma. Approaches that capture plasma on paper (if testing is not then performed on that paper) require extraction of the plasma from the paper, requiring drying time and elution steps that are not currently appropriate for POC testing. Unfortunately, existing stand-alone devices capable of generating liquid plasma from capillary blood generate plasma volumes that are relatively small. For example, the A-PON Plasma Separator device (53) collects blood via a capillary collection tube coated with dried anticoagulant; blood then passes through a porous membrane that separates blood cells and platelets. Plasma collects in the device’s plasma reservoir and is dispensed from the device when the operator pushes an air-filled bulb. While satisfying the goals of separation of liquid plasma in a short time frame (approximately 5 minutes) and yielding a consistent volume of plasma (useful for quantitative testing), sample input volume and plasma recovery are low (14 µL plasma recovered from a 75 µL capillary WB sample) (53). The Plasma Separator device from CanaryQ (54) also uses filtration to separate an anticoagulated 100 µL sample (collected separately and dispensed into the device with a compatible tool), generating approximately 30 µL of plasma in approximately 1 minute. The PlasmaDrop PD-50 system also appears to use a separation membrane (described as an “RBC retentive medium”); while this device can accommodate a larger starting blood volume, plasma yield is quite low (55). Other interesting approaches to liquid plasma generation via passive separation techniques with an eye towards POC use have been evaluated on a research basis (52) but are not commercially available.

Approaches to plasma generation that are based on selective depletion of red blood cells (e.g., see reference 56) introduce both known and unknown assay validation complications due to the requirement to measure specific analytes in the presence of retained white blood cells (WBCs) and platelets. In addition to creating new requirements for assay validation, retained WBC may in some cases impact the actual measurement, such as proviral DNA that can confound measurement of HIV RNA (57).

### User limitations and user experience

In a recent prospective field evaluation of the Xpert HCV test in which we authors participated (58), it was noted that rates of successful collection of the ≥250 µL FS sample into the BD Microtainer improved with practice. Consistent hand warming (using a warming pad even until the hand felt slightly uncomfortable) and use of a high-flow lancet (BD contact-activated lancet, catalog #366594) were found to be critical to yield adequate volume. With these measures in place, 500 µL was easily obtained from one FS, and in some instances, we were able to obtain as much as 1.5 mL from a single FS. Experience also helped, as operators learned where to apply the lancet and how to position the finger so that maximum flow was obtained and both the donor and person conducting the FS were comfortable. Notably, the time for collection of a 250 µL volume was substantial (typically 10–15 minutes, including handwarming). In an additional informal FS collection study run for a separate research project by one of us authors, it was observed that watching the Xpert HCV instructional video (13) was sufficient to allow naive operators to successfully collect the 250 µL minimum volume into the BD microtainer. Notably, these collections were performed without the BD MiniDraw apparatus.

While a small learning curve may be required to achieve consistent success with these large-volume capillary blood collection approaches, many studies have examined the patient or user experience with these new devices. Generally, these studies have found that adult users more often prefer the novel device over venous sampling, which was examined with the BD MiniDraw (59), Tasso+ (31, 60–62), and Tap Micro (60). In a study examining its potential role in STI and HIV testing, approximately 30% preferred Tasso+, but another 40% were equivocal on a preference between Tasso+ or venipuncture, while slightly less than 30% still preferred venipuncture by a nurse (27).

Experience with novel collection devices and children also appears to be limited. Regulatory approval specifies that the BD MiniDraw is cleared only for adults age 18 and older (15, 16), while the other devices do not specify age in FDA summaries. Boffel et al. performed a study that included the Tasso+ in adolescents aged 13–17 years (63). The user experience with the device was generally positive, but analytical assessment was limited.

### Cost

A critical barrier to the use of large-volume capillary blood production/collection devices is cost. While collection of FS samples directly into a microtainer is a relatively cost-effective option, with lancets costing ~$0.25 and K2EDTA microtainer tubes costing ~$1.25, additional tools to facilitate FS collection (e.g., BD MiniDraw apparatus) and arm-based collection devices are relatively expensive (from approximately $10 if purchased in bulk to more than $50 for an arm-based device kit purchased online). Recognizing this barrier, work is in progress to develop simpler solutions that lower cost but maintain the benefits related to user experience, volume captured, and sample quality (45).

## OPPORTUNITIES AND CHALLENGES SPECIFIC TO CLINICAL MICROBIOLOGY TESTING

As evidenced by the transformative example of the POC Xpert HCV test, large-volume capillary blood collection offers the opportunity to decentralize clinical microbiology testing through application to blood-based POC and ultimately home use tests. Notably, this approach also offers the opportunity to facilitate phlebotomy-free collection of samples for testing in centralized laboratories, increasing accessibility to medium and high complexity testing. In particular, there are many opportunities for impactful applications in molecular testing, such as quantitative HIV RNA, HCV RNA, and CMV DNA measurements. As discussed earlier, collection of a large capillary blood volume also offers opportunities for performing multiple assays (potentially including both molecular and serological tests, assuming matrix compatibility) from a single specimen, as illustrated by examples in STI screening, PrEP monitoring, and HIV monitoring. In many cases, more work will be needed to validate both specimen stability (over the relevant transport and storage conditions) and the impact of hemolysis on analyte measurement for large-volume capillary samples.

One potential barrier to adopting large-volume capillary sampling for clinical microbiology testing remains sample volume. Although the new devices discussed in this review enable more consistent collection of larger volumes of blood relative to traditional fingerstick, they still yield significantly less blood than traditional venipuncture. Notably, the required sample volumes for many clinical microbiology tests (particularly those performed on automated instruments) exceed the amount actually used by the instrument for analysis, with much of the sample volume constituting unused “dead volume.” Manufacturers of these lab-based instruments should consider the opportunities inherent in large-volume capillary sampling and strive to reduce dead volume requirements in the next generation of technology. Moreover, improving the ability to use a single specimen volume for multiple tests on one analyzer (e.g., through use of a shared nucleic acid extract) would also help maximize the utility of a given specimen volume. If this can be accomplished, the impact on performance of using a large-volume capillary sample vs a standard volume venous sample may be minimal, thus allowing capillary sampling to replace venipuncture in many testing applications.

In the meantime, the common approach to overcoming insufficient sample volume for testing with higher-complexity assays is dilution, as was utilized in the previously discussed study of CMV viral load by Phan et al. (26). Sample dilution may introduce inaccuracy, especially for quantitative tests, and will impact detection for samples with low concentrations of analyte. More studies are needed to clarify the impact of sample dilution across clinical microbiology testing contexts. For example, very little capillary-venous discordance was observed in the CMV study; the only samples that were not detected by capillary testing had very low viral load, which likely has minimal clinical impact on the management of CMV. For HIV viral load, as another example, where clinically significant differences manifest as order of magnitude changes in viral load, a measurement within 0.5 log of the actual value may be sufficient to properly inform clinical decision-making in most cases. For some viruses, inability to detect viral loads below ~1,000 copies/mL may have minimal impact on clinical decision-making in some use cases even though state-of-the-art assays using venous blood are sometimes orders of magnitude more sensitive (48, 64).

Finally, a remaining barrier to leveraging phlebotomy-free large-volume capillary blood sampling for clinical microbiology is the need for general clearance of these tools for measurement of molecular analytes. The questions raised in the preceding discussion regarding how currently cleared devices can or cannot be used and what additional validation data are needed to achieve certain claims (e.g., general use for molecular testing for a particular virus) must be answered in order to facilitate adoption of large-volume capillary sampling approaches for clinical microbiology testing across testing settings.

## CONCLUSION

The validation of a large-volume FS sample for the newly FDA-authorized Xpert HCV test has created an important precedent for the use of a large-volume capillary blood sample for POC testing. This advance opens the door to the development and deployment of a wide range of POC tests utilizing large-volume capillary samples, including tests that require larger sample volumes for detection of low-concentration analytes. Multiple tools for production and collection of large-volume capillary samples now exist, most of which yield blood that demonstrates excellent concordance with venous blood across a range of analytes, though analytes like potassium and platelets remain challenging for the arm-based tools and data for molecular analytes are limited overall. As the implementation of these devices in POC sampling and ultimately self-sampling proceeds, the relative strengths and limitations of each device will have to be established. Furthermore, understanding the regulatory expectations for pairing large-volume sample production tools and collection containers with POC testing devices will facilitate both uptake of large-volume collection strategies and POC blood test development, particularly because allowing multiple POC tests to be performed from one large-volume capillary sample would substantially improve the patient experience. Tools capable of separating liquid plasma from large-volume capillary whole blood samples for POC testing remain an elusive but critical goal. A recent funding challenge from the Gates Foundation for the development of phlebotomist-free, large-volume whole blood collection devices (65) substantiates the current and future importance of large-volume capillary blood collection for global health diagnostics, including those performed at the POC.
